# Supplementary material for: Was the Reduction in Seasonal Influenza Transmission during 2020 Attributable to Non-Pharmaceutical Interventions to Contain Coronavirus Disease 2019 (COVID-19) in Japan?
Source: Viruses. 2022 Jun 28;14(7):1417. doi: 10.3390/v14071417 (PMC9320739; doi:10.3390/v14071417)
Supplement: Supplementary file 1 [file viruses-14-01417-s001.zip › viruses-1716755-supplementary.pdf]

Supplementary Files for: Was the Reduction in Seasonal Influenza Transmission Attributable to Non-pharmaceutical Interventions to Contain Coronavirus Disease 2019 (COVID-19) in Japan?

Authors:
Keita Wagatsuma[1,2], Iain S. Koolhof[3], & Reiko Saito[1]

Author affiliations:
[1] Division of International Health (Public Health), Graduate School of Medical and Dental Sciences, Niigata University, Niigata 951-8510, Japan
[2] Japan Society for the Promotion of Science, Tokyo 102-0083, Japan
[3] College of Health and Medicine, School of Medicine, University of Tasmania, Hobart 7000, Australia

Corresponding author:
Keita Wagatsuma
Division of International Health (Public Health)
Graduate School of Medical and Dental Sciences, Niigata University
1-757 Asahimachi-dori, Chuo-ku, Niigata City, Niigata 951-8510, Japan
Tel: +81-25-227-2129; Fax +81-25-227-0765
E-mail address: waga@med.niigata-u.ac.jp

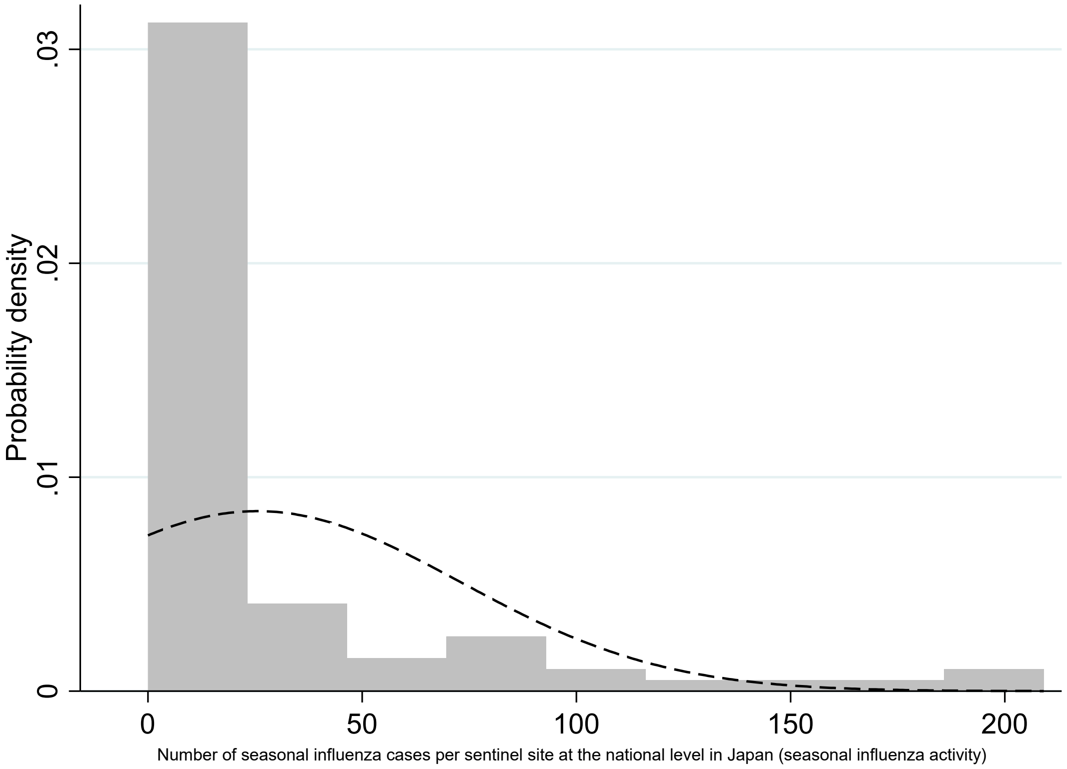

Figure S1. Probability distribution of the monthly seasonal influenza activity at the national level in Japan during 2014–2020. The mean monthly total number of influenza cases per sentinel site at the national level in Japan was 25.4 (standard deviation [SD], 47.4). These observational data do not follow a normal distribution (Shapiro-Wilk test, p<0.001).

Table S1. Assessments of multicollinearity using pairwise Spearman's rank-order cross correlation coefficients of independent variables at the national level in Japan during 2014–2020

Independent Variable	1	2	3	4	5
1. Retail sales of hand hygiene products [a]	1.00				
2. Domestic airline passenger arrivals [b]	–0.18	1.00			
3. International airline passenger arrivals [c]	–0.13	0.70***	1.00		
4. Average ambient temperature	0.07	0.35***	0.07	1.00	
5. Relative humidity	0.15	0.25*	0.03	0.66*	1.00

[a] Retail sales of hand hygiene products per ¥1 billion (units: yen) at the national level in Japan during 2014–2020. [b] Number of domestic airline passenger arrivals per one million population (units: person) at the national level in Japan during 2014–2020. [c] Number of international airline passenger arrivals per one million population (units: person) at the national level in Japan during 2014–2020. The significant predictors are described by *p<0.05, **p<0.01, ***p<0.001.

Table S2. Optimal delayed effect selection of statistical model for individual meteorological conditions according to AIC

Independent Variable	Units	AIC				
		Single lag (months)				
		0	1	2	3	4
Average ambient temperature	°C	5.43	5.86	7.40	8.32	7.62
Relative humidity	%	6.90	7.92	8.38	7.92	7.72
Independent variable	Units	AIC				
		Cumulative lag (months)				
		0–0	0–1	0–2	0–3	0–4
Average ambient temperature	°C	NA	5.41	5.77	6.44	7.28
Relative humidity	%	NA	7.14	7.68	8.14	8.33

Abbreviations: NA: Not available, AIC: Akaike Information Criterions. Notes: The standard time-series generalized linear regression models (GLMs) with gamma distribution and logarithmic link function with robust error variances between the monthly total number of seasonal influenza cases per sentinel site at the national level in Japan and single variables of meteorological conditions (average ambient temperature and relative humidity) with single and cumulative lags of 0–4 months were assessed based on AIC for model calibration. Generally, lower AIC indicates a better statistical model fit. Based on AIC, the optimal delayed effect for average ambient temperature and relative humidity were 0–1 and 0 months, respectively.

Table S3. Descriptive statistics of monthly seasonal influenza activity, alternative indicators of NPIs, and meteorological conditions at the national level in Japan by year during 2014–2020

Variable	Units	Mean (SD)						
		2014	2015	2016	2017	2018	2019	2020
Seasonal influenza activity [a]	Cases per sentinel site	29.30 (41.44)	19.75 (46.12)	29.42 (53.34)	27.08 (41.45)	31.92 (62.57)	31.50 (60.23)	9.51 (23.87)
Retail sales of hand hygiene products [b]	Yen	6.66 (0.61)	6.90 (0.62)	7.12 (0.95)	7.27 (0.77)	7.32 (0.58)	7.39 (0.72)	10.98 (1.97)
Domestic airline passenger arrivals [c]	Person	7.87 (0.79)	8.04 (0.72)	8.10 (0.73)	8.47 (0.78)	8.59 (0.72)	8.89 (0.70)	3.89 (2.37)
International airline passenger arrivals [d]	Person	1.33 (0.08)	1.49 (0.09)	1.70 (0.08)	1.82 (0.08)	1.94 (0.08)	1.95 (0.08)	0.36 (0.63)
Average ambient temperature	°C	16.46 (8.09)	17.02 (7.51)	17.34 (7.97)	16.55 (8.33)	17.12 (8.40)	17.32 (7.85)	17.35 (7.75)
Relative humidity	%	68.98 (5.49)	70.19 (5.38)	70.48 (4.89)	69.19 (5.33)	70.21 (4.56)	70.16 (6.38)	71.31 (5.67)

Abbreviations: SD: Standard deviation. [a] Total number of seasonal influenza cases per sentinel site at the national level in Japan during 2014–2020. [b] Retail sales of hand hygiene products per ¥1 billion (units: yen) at the national level in Japan during 2014–2020. [c] Number of domestic airline passenger arrivals per one million population (units: person) at the national level in Japan during 2014–2020. [d] Number of international airline passenger arrivals per one million population (units: person) at the national level in Japan during 2014–2020. Notes: The mean, together with the range of independent and dependent variables, are described by year.

Table S4. Time-series associations between seasonal influenza activity and alternative indicators of NPIs adjusted for meteorological conditions with optimal delayed effects, seasonality and cycle, and autocorrelation term at the national level in Japan during 2014–2020

Independent Variable	Logarithmic of Adjusted Linear Regression Coefficients per 1-unit Change of Each Individual Variable (95% CI)				
	Single lag (months)				
	0	1	2	3	4
Retail sales of hand hygiene products [a]	–0.46*** (–0.68, –0.24) AIC=4.55 α=0.85	–0.55*** (–0.85, –0.26) AIC=4.58 α=1.11	–0.45 (–0.97, 0.06) AIC=4.59 α=1.62	–0.70*** (–1.08, –0.32) AIC=4.34 α=0.93	–0.50*** (–0.78, –0.23) AIC=4.41 α=1.10
Domestic airline passenger arrivals [b]	0.63*** (0.33, 0.92) AIC=4.44 α=1.03	0.88*** (0.45, 1.31) AIC=4.25 α=1.04	0.71*** (0.43, 0.98) AIC=4.17 α=0.92	0.64*** (0.32, 0.96) AIC=4.27 α=1.04	0.53*** (0.26, 0.80) AIC=4.28 α=0.94
International airline passenger arrivals [c]	2.24*** (1.68, 3.16) AIC=4.39 α=0.99	2.93*** (1.95, 3.91) AIC=4.21 α=0.82	2.57*** (1.62, 3.52) AIC=4.18 α=0.90	2.22*** (1.18, 3.26) AIC=4.25 α= 0.99	2.05*** (1.08, 3.02) AIC=4.27 α=0.89
Independent Variable	Logarithmic of Adjusted Linear Regression Coefficients per 1-unit Change of Each Individual Variable (95% CI)				
	Cumulative lag (months)				
	0–0	0–1	0–2	0–3	0–4
Retail sales of hand hygiene products [a]	NA (NA, NA) AIC=NA α=NA	–0.78*** (–1.04, –0.52) AIC=4.44 α=0.76	–1.20*** (–1.61, –0.79) AIC=4.23 α=0.95	–1.55*** (–2.00, –1.09) AIC=4.01 α=0.95	–1.48*** (–1.98, –0.99) AIC=4.00 α=1.00
Domestic airline passenger arrivals [b]	NA (NA, NA) AIC=NA	1.09*** (0.64, 1.54) AIC=4.18	1.16*** (0.67, 1.65) AIC=3.99	1.06*** (0.64, 1.49) AIC=3.97	0.95*** (0.59, 1.32) AIC=3.98

	α=NA	α=0.96	α=1.12	α=1.11	α=1.04
International airline passenger arrivals [c]	NA	3.01***	3.09***	2.96***	2.78***
	(NA, NA)	(2.04, 3.98)	(2.09, 4.09)	(1.97, 3.94)	(1.85, 3.72)
	AIC=NA	AIC=4.25	AIC=4.12	AIC=4.06	AIC=4.06
	α=NA	α=0.88	α=0.89	α=0.93	α=0.92

[a] Retail sales of hand hygiene products per ¥1 billion (units: yen) at the national level in Japan during 2014–2020. [b] Number of domestic airline passenger arrivals per one million population (units: person) at the national level in Japan during 2014–2020. [c] Number of international airline passenger arrivals per one million population (units: person) at the national level in Japan during 2014–2020. Abbreviations: NA: Not available, CI: Confidence interval, AIC: Akaike information criterion, α: Dispersion parameter. Note: Table S3 presents the estimates in Figure 2. The logarithmically adjusted linear regression coefficients (together with 95% CIs) are the per 1-unit change in alternative indicators of non-pharmaceutical interventions (NPIs). Each estimate of the statistical model also includes AIC and α. Different lag structures in the associations between seasonal influenza activity and NPI indicators were also explored using single lag months (lag 0, 1, 2, 3, and 4 months) and cumulative lag months (lag 0–1, 0–2, 0–3, and 0–4 months moving average). The standard time-series generalized linear regression models (GLMs) with gamma distribution and logarithmic link function with robust error variances were adjusted for meteorological conditions with optimal delayed effects (i.e., average ambient temperature at lag 0–1 month and relative humidity at 0 months), year variables (2014, 2015, 2016, 2017, 2018, 2019, and 2020), and autocorrelation terms. Detailed descriptions of the statistical modeling inference are provided in the Materials and Methods section of the main manuscript. The significant predictors in the statistical model are indicated by $^{*}p<0.05$, $^{**}p<0.01$, $^{***}p<0.001$.

Table S5. Time-series associations between seasonal influenza activity and alternative indicators of NPIs adjusted for meteorological conditions with optimal delayed effects, seasonality and cycle, and autocorrelation term at the national level in Japan during 2014–June 2021

Independent Variable	Logarithmic of Adjusted Linear Regression Coefficients per 1-unit Change of Each Individual Variable (95% CI)				
	Single lag (months)				
	0	1	2	3	4
Retail sales of hand hygiene products [a]	–0.37**<br>(–0.61, –0.13)<br>AIC=3.70<br>α=0.88	–0.40**<br>(–0.70, –0.11)<br>AIC=3.72<br>α=1.13	–0.27<br>(–0.79, 0.24)<br>AIC=3.71<br>α=1.46	–0.53*<br>(–0.96, –0.10)<br>AIC=3.53<br>α=0.99	–0.37*<br>(–0.71, –0.04)<br>AIC=3.55<br>α=1.16
Domestic airline passenger arrivals [b]	0.60***<br>(0.31, 0.89)<br>AIC=3.55<br>α=1.06	0.74***<br>(0.35, 1.12)<br>AIC=3.45<br>α=1.04	0.63***<br>(0.38, 0.89)<br>AIC=3.31<br>α=0.99	0.66***<br>(0.35, 0.96)<br>AIC=3.30<br>α=1.08	0.54***<br>(0.27, 0.80)<br>AIC=3.33<br>α=0.98
International airline passenger arrivals [c]	2.40***<br>(1.66, 3.13)<br>AIC=3.48<br>α=1.00	2.82***<br>(1.88, 3.76)<br>AIC=3.33<br>α=0.88	2.51***<br>(1.58, 3.44)<br>AIC=3.28<br>α=0.94	2.17***<br>(1.15, 3.18)<br>AIC=3.34<br>α=1.01	1.98***<br>(1.02, 2.94)<br>AIC=3.35<br>α=0.92

Independent Variable	Logarithmic of Adjusted Linear Regression Coefficients per 1-unit Change of Each Individual Variable (95% CI)				
	Cumulative lag (months)				
	0–0	0–1	0–2	0–3	0–4
Retail sales of hand hygiene products [a]	NA<br>(NA, NA)<br>AIC=NA<br>α=NA	–0.63***<br>(–0.94, –0.33)<br>AIC=3.62<br>α=0.79	–1.00***<br>(–1.48, –0.51)<br>AIC=3.46<br>α=0.98	–1.26***<br>(–1.85, –0.67)<br>AIC=3.30<br>α=0.90	–1.19***<br>(–1.80, –0.59)<br>AIC=3.28<br>α=0.91
Domestic airline passenger arrivals [b]	NA<br>(NA, NA)	1.02***<br>(0.58, 1.45)	1.05***<br>(0.60, 1.49)	1.00***<br>(0.60, 1.40)	0.94***<br>(0.58, 1.29)

	AIC=NA	AIC=3.34	AIC=3.17	AIC=3.09	AIC=3.07
	α=NA	α=0.98	α=1.12	α=1.16	α=1.10
International airline passenger arrivals [c]	NA	2.93***	2.99***	2.86***	2.69***
	(NA, NA)	(1.98, 3.89)	(2.02, 3.97)	(1.90, 3.82)	(1.78, 3.61)
	AIC=NA	AIC=3.37	AIC=3.23	AIC=3.17	AIC=3.16
	α=NA	α=0.91	α=0.93	α=0.97	α=0.95

[a] Retail sales of hand hygiene products per ¥1 billion (units: yen) at the national level in Japan from 2014 to June 2021. [b] Number of domestic airline passenger arrivals per one million population (units: person) at the national level in Japan during 2014–June 2021. [c] Number of international airline passenger arrivals per one million population (units: person) at the national level in Japan from 2014 to June 2021. Abbreviations: NA: Not available, CI: Confidence interval, AIC: Akaike information criterion, α: Dispersion parameter. Notes: Logarithms of adjusted linear regression coefficients (together with 95% CIs) are per 1-unit change in alternative indicators of non-pharmaceutical interventions (NPIs). Each estimate of the statistical model also includes AIC and α. Different lag structures in the associations between seasonal influenza activity and NPI indicators were also explored using single lag months (lag 0, 1, 2, 3, and 4 months) and cumulative lag months (lag 0–1, 0–2, 0–3, and 0–4 months moving average). The standard time-series generalized linear regression models (GLMs) with gamma distribution and logarithmic link function with robust error variances were adjusted for meteorological conditions with optimal delayed effects (i.e., average ambient temperature at lag 0–1 month and relative humidity at lag 0 month), year variables (2014, 2015, 2016, 2017, 2018, 2019, 2020, and 2021), and autocorrelation terms. Detailed descriptions of the statistical modeling inference are provided in the Materials and Methods section of the main manuscript. The significant predictors in the statistical model are indicated by $^{*}p<0.05$, $^{**}p<0.01$, $^{***}p<0.001$.
